# What predicts doctors’ satisfaction with their chosen medical specialty? A Finnish national study

**DOI:** 10.1186/s12909-016-0643-z

**Published:** 2016-04-26

**Authors:** Teppo J. Heikkilä, Harri Hyppölä, Jukka Vänskä, Hannu Halila, Santero Kujala, Irma Virjo, Markku Sumanen, Elise Kosunen, Kari Mattila

**Affiliations:** Unit of Primary Health Care, Hospital District of Northern Savo, P.O. Box 1777, FI 70211 Kuopio, Finland; Emergency Department, Kuopio University Hospital, P.O. Box 1777, FI 70211 Kuopio, Finland; Finnish Medical Association, P.O. Box 49, FI 00501 Helsinki, Finland; Department of General Practice, School of Medicine, University of Tampere, Kalevantie 4, FI 33014 Tampere, Finland; Centre of General Practice, Pirkanmaa Hospital District, P.O. Box 2000, FI 33521 Tampere, Finland

## Abstract

**Background:**

In Finland the number of medical specialists varies between specialties and regions. More regulation of the post-graduate medical training is planned. Therefore, it is important to clarify what predicts doctors’ satisfaction with their chosen specialty.

**Methods:**

A random sample contained 50 % of all Finnish doctors under 70 years of age. The respose rate was 50.5 %. Working-age specialists were asked to value their motives when choosing a specialty. They were also asked if they would choose the same specialty again. The odds ratios for not choosing the same specialty again were tested.

**Results:**

*Diversity of work* was the most important motive (74 % of respondents). Seventeen percent of GPs would not choose the same specialty again, compared to 2 % of ophthalmologists and 4 % of pediatricians. A major role of *Diversity of work* and *Prestigious field* correlated with satisfaction whereas *Chance* with dissatisfaction with the specialty.

**Discussion:**

Motives and issues related to the work and training best correlate with satisfaction with the specialty.

**Conclusions:**

When the numbers of Finnish postgraduate medical training posts become regulated, a renewed focus should be given to finding the most suitable speciality for each doctor. Information about employment and career advice should play an important role in this.

## Background

As the population – and also health care personnel – are ageing, major challenges will emerge in meeting the need of equal health care services for the entire population [1–3]. In Finland it was recently found that the problems concerning a sufficient number of medical specialists in the future are going to vary greatly in different medical specialties and also in different regions [4, 5]. For example, psychiatric disciplines seem to have problems in having enough specialists in the whole Finland while some surgical specialties are at risk of excess of specialists at least in some areas. One of the main reasons for this workforce imbalance is that in Finland, in practice, a doctor has been able to choose the specialty he or she prefers without any restrictions.

It has been found that an interest in people is the most important factor when a young student is entering medicine [6, 7]. Furthermore, the content of the work also seems to direct the choice of specialty during studies [8]. However, the idea of future medical specialty is not stable during undergraduate studies and it can be seen as a process that evolves during medical training [9–11]. Even after that, the choice of medical specialty is not always stable and the stability of the choice also varies between specialties [12].

The career satisfaction of a doctor is a complex question. It may be affected by, for example, workload, workplace stress, organisation of the work, quality of care and ability to access quality services for patients and fair distribution of rewards [13–22]. There are also differences between specialties in the importance of different factors explaining job satisfaction [22, 23].

In Finland, 60 % of all working-age doctors and 81 % of working-age doctors over 45 years old are medical specialists [24, 25]. Of all specialists, 24 % have two or more specialties (Finnish Medical Association, unpublished information). Females comprise 59 % of all working-age doctors and 57 % of medical specialists [26]. Only 1 % of young Finnish doctors do not intend to specialize [27].

Because of the imbalance of medical specialists noted recently, there are now plans to develop a new selection process for postgraduate medical training so that the imbalance can be corrected [28]. This is a part of other plans stated after a nearly decade-long debate about developing postgraduate medical and dental education in Finland [28–30].

In this present situation in Finland, it is important to find out how medical graduates can be directed to choose specialties where there is shortage of specialists in a way that ensures that they are also motivated and will stay in that particular career path. The aim of this study was to find out what the main reasons are for choosing a medical specialty and whether there are any correlations between these motives and dissatisfaction with the chosen specialty.

## Methods

The Physician 2013 study was undertaken as a collaborative project of the University of Eastern Finland (formerly University of Kuopio), the University of Tampere and the Finnish Medical Association [31]. It followed previous studies conducted in 1988, 1993, 1998, 2003 and 2008. The study compiled information on social background, work history, labour market and career plans in the medical profession in Finland. It also assessed doctors’ views of undergraduate and specialist training, values and professional identity. The questions were mostly formed before the first study in 1988, although some new questions have been added in later questionnaires. Most of the questions used in the inquiry have existed in the same form since the first questionnaire, for reasons of comparability. In the 2013 study, both postal and electronic questionnaires were used. Addresses were collected from the database of the Finnish Medical Association, which has details on all doctors licensed in Finland. The basic study population in the Physician 2013 study was comprised of all Finnish doctors under 70 years of age (*N* = 21,501). A random sample of approximately 50 % was drawn from this basic study population based on the subjects’ birthdays so that only those born on odd-numbered days were selected for the sample (*n* = 10,600). The formation of the data is presented in Table 1.

**Table 1:** Forming the data of the Physician 2013 study

Study population	21,501
Study sample	10,600
Returned questionnaires	
- Email	2148
- Posted	3202
- In total	5350
Response rate (%)	50.5

The response rate of women (53 %) was higher than the response rate of men (46 %). The response rate varied in different age groups, being the lowest in the group of 35–44-year-old respondents. Medical specialists (55 %) also answered more often than unspecialised doctors (43 %). To control possible non-response bias and to improve the representativeness of the results, age, gender and specialization status distributions of all Finnish doctors were used to calculate weights to each survey respondent. People in under-represented groups were given a weight greater than 1 and those in over-represented groups were given a weight smaller than one, with the weighting being proportionate to the degree of over- or under-representation.. The distributions concerning all Finnish doctors were derived from the register of Finnish Medical Association. For the analysis of this study, the working-age medical specialists were selected from the weighted data.

The respondents were asked: *“If you are a specialist or in specialist training, to what extent did the following items affect your choice of specialty?”* and they were presented with eleven items which could have influenced their choice. This same question has been asked in the previous studies. The data were classified by means of a Likert five-point scale. The respondents were also asked: *“If you were making the choice again, would you still choose the same medical specialty?”*

The respondents were grouped based on gender, age, working sector, specialty and university of specialist training. The data were analysed using cross-tabulation and a *Chi-squared test* to test differences between different groups of doctors if they would have chosen a different specialty if making the choice again. To calculate odds ratios (with 95 % confidence intervals) for the risk of answering *“No”* to the question *“If you were making the choice again, would you still choose the same medical specialty?”* a binary logistic regression model was also made with gender, age, items named as important motives for choosing a specialty by more than 25 % of the respondents, correspondence between specialist training and current work, working sector and specialty as independent variables. *Nagelkerke’s R-squared* and *Hosmer-Lewenshow tests* were conducted for the logistic regression model. The data were analysed using IBM SPSS 22.0.0.0 for Macintosh predictive analytics software.

## Results

**Fig. 1:**
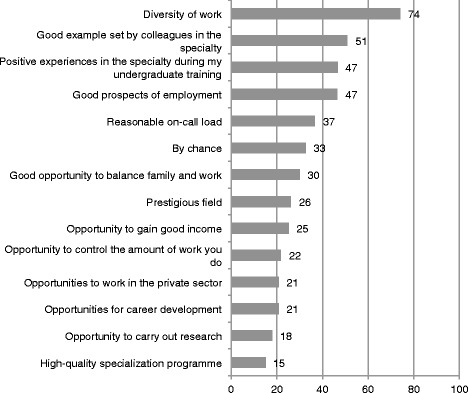
Motives to choose a medial specialty in Physician 2013 Study. Proportions (%) of working-age medical specialists who answered *“Considerably”* or *“Very much”* to the question *“If you are a specialist or in specialist training, to what extent did the following items affect your choice of specialty?”* in Physician 2013 study (*n* = 2796)

The most frequent motive for choosing a specialty was *Diversity of work*, followed by *Good example set by colleagues in the specialty*, *Positive experiences in the specialty during undergraduate training* and *Good prospects of employment* (Fig. 1).

**Fig. 2:**
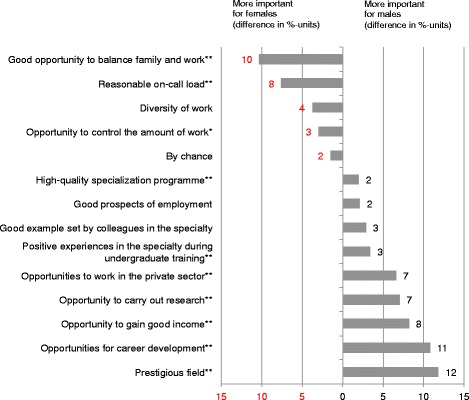
Differences between male and female doctors when choosing a medical specialty. Differences of proportions (%-units) of working-age male and female medical specialists who answered *“Considerably”* or *“Very much”* to the question *“If you are a specialist or in specialist training, to what extent did the following items affect your choice of specialty?”* in Physician 2013 study. **p <0.01, *p <0.05, males *n* = 1256 and females *n* = 1540

When the answers of male and female respondents were compared, there were some significant differences in the motives for choosing a medical specialty (Fig. 2). *Prestigious field*, *Opportunities for career development*, *Opportunity to gain a good income*, *Opportunity to carry out research*, *Opportunities to work in the private sector* and *Positive experiences in the specialty during undergraduate training* were significantly more important motives for male respondents. On the other hand, *Good opportunity to balance family and w*ork, *Reasonable on-call load* and *Opportunity to control the amount of work* were more important for female respondents.

Of all the respondents, 12 % would not have chosen the same specialty if making the choice again (Table 2). There was no significant difference between genders. Older doctors would not have chosen the same specialty more often compared with younger doctors.

**Table 2:** Proportions of those respondents who would not choose the same medical specialty again

			Answered “*No*”
		*n*	%
*Gender*	Males	1174	12.1
	Females	1438	11.9
Age**	Under 45 y. o.	958	6.8
	45–54 y. o.	936	13.3
	55–64 y. o.	717	14.5
Working sector*	Specialized medical care	1299	10.7
	Primary health care	392	11.5
	Public institutions	172	14.0
	Private sector	656	13.1
*University of specialist training*	Helsinki	834	12.9
	Kuopio	324	12.3
	Oulu	427	13.8
	Tampere	523	9.9
	Turku	365	9.9
	Foreign university	37	10.8
Specialty**	General Practice	395	17.4
	Anaesthesiology and intensive care medicine	196	16.3
	Other Specialties	390	14.9
	Psychiatry	284	14.1
	Occupational Health	178	12.9
	Otorhinolaryngology	79	10.1
	Obstetrics and gynaecology	139	9.2
	Radiology	99	9.1
	Internal medicine	243	9.0
	Surgery	298	8.7
	Neurology	61	6.6
	Paediatrics	155	4.5
	Ophthalmology	90	2.2
*All together*		2612	12.0

Proportions (%) of the respondents answering *“No”* to the question *“If you were making the choice again, would you still choose the same medical specialty?”* of working-age medical specialists by gender, age, working sector, specialty, and university of specialist training in Physician 2013 study. *Specialized medical care*: university hospital, other public hospital. *Primary health care*: health centre, public occupational health care. *Public institutions*: government agency or institution, university. *Internal medicine*: cardiology, clinical haematology, endocrinology, gastroenterology, infectious diseases, internal medicine, nephrology, rheumatology. *Surgery*: cardiothoracic surgery, gastroenterological surgery, general surgery, hand surgery, oral and maxillofacial surgery, orthopaedics and traumatology, paediatric surgery, plastic surgery, urology, vascular surgery. *Psychiatry*: adolescent psychiatry, child psychiatry, forensic psychiatry, psychiatry. *Other specialties*: child neurology, clinical chemistry, clinical genetics, clinical microbiology, clinical neurophysiology, clinical pharmacology and pharmacotherapy, clinical physiology and nuclear medicine, dermatology and allergology, forensic medicine, geriatrics, oncology, pathology, phoniatrics, physical and rehabilitation medicine, public health, respiratory medicine and allergology, sports medicine. **p <0.01, *p <0.05

The differences between doctors working in different working sectors were rather small, but statistically significant. A smaller proportion of doctors working in specialised medical care answered *“No”* to the question *“If you were making the choice again, would you still choose the same medical specialty?”* compared with the doctors in primary health care, the private sector and public institutions.

Almost one-fifth of specialists in general practice and anaesthesiologists would choose a different specialty if making the choice again, while only fewer than 5 % of paediatricians and a little over 2 % of ophthalmologists were not satisfied with their choice of specialty. There were no statistically significant differences between universities of specialist training among those who would not choose the same specialty again.

In the binary logistic regression model, when odds ratios for the risk of answering *“No”* to the question *“If you were making the choice again, would you still choose the same medical specialty?”* were calculated, there was no statistical difference between genders. The age groups of 45–54-year-old and 55–64-year-old respondents had a higher odds ratio to answer *“No”* compared with the under-45-year-old respondents (Table 3).

**Table 3:**
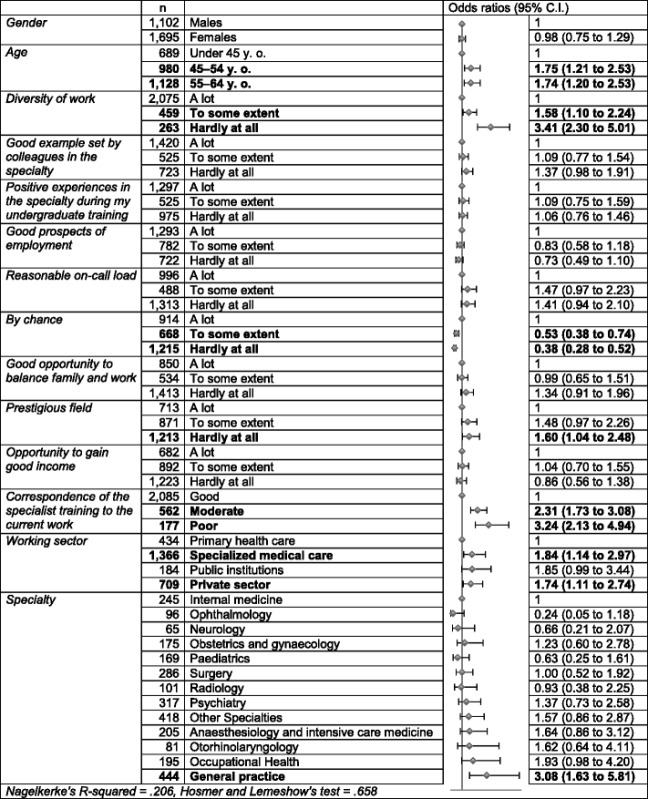
Odds ratios for not choosing the same medical specialty again

Odds ratios in binary logistic regression model with 95 % confidence interval for working-age medical specialists answering *“No”* to the question: “*If you were making the choice again, would you still choose the same medical specialty?*” in Physician 2013 study. Statistically significant (*p* <0.05) values are in BOLD

When looking at the different motives affecting the choice of a medical specialty, the only differences came in *Diversity of work*, *By chance* and *Prestigious field*. The respondents who felt that *Diversity of work* and *Prestigious field* were important motives for choosing a specialty had a lower odds ratio to answer *“No”* to the question *“If you were making the choice again, would you still choose the same medical specialty?”* compared with the respondents who regarded these motives less important. The respondents who felt *By chance* was an important motive had a higher odds ratio to answer *“No”* than the others.

The respondents who reported *Good correspondence between specialist training and current work* had a significantly lower odds ratio to answer *“No”* to the question *“If you were making the choice again, would you still choose the same medical specialty?”* compared with the other respondents. Doctors working in primary health care had a significantly lower odds ratio to answer *“No”* compared with doctors working in specialized medical care and in the private sector.

General practitioners’ odds ratio to answer *“No”* to the question *“If you were making the choice again, would you still choose the same medical specialty?”* was significantly higher compared with internists, ophthalmologists and paediatricians.

## Discussion

According to this study, the motive to choose one’s medical specialty that best correlated with satisfaction with the chosen medical specialty was *Diversity of work*. Another main finding was that *Correspondence between specialist training and current work* had a significant correlation with satisfaction with the specialty. A major role of *Chance* in selection of the specialty correlated with dissatisfaction with the specialty. Motives of males and females to choose a specialty differed significantly.

*Diversity of work* was the main motive for choosing a specialty, as it was also in the previous Physician 2008 study [32]. However, medical students’ first experiences of colleagues and the content of the particular specialty also seem to have a major role in selection of the specialty [3, 32–36]. It is also noteworthy, although natural, that doctors want to evaluate possibilities for future employment when choosing a medical specialty.

In this study, 12 % of the respondents would not have chosen the same medical specialty if making the choice now. This means that a large majority of the specialists were actually quite happy with their choice. Still, even though the proportion of dissatisfied specialists was rather small, it was notable. Also, it has to be noted that there were some significant differences between specialties in this matter, revealing that despite the quite good overall situation, there are some specialties that would need some attention. Specialists in general practice, in particular, had a significant odds ratio to be dissatisfied with their medical specialty compared with some other specialties. On the other hand, working in primary health care reduced the odds ratio of dissatisfaction with the specialty. At first glance there seems to be a discrepancy in these findings, since when examined independently, respondents working in primary health care were somewhat more dissatisfied with their specialty than those working in specialised medical care. One explanation for this might be that in Finland medical specialists, especially specialists in general practice, work in many different fields of medicine, and therefore also satisfaction with the specialty may vary accordingly. For example, approximately 30 % of Finnish specialists in general practice work outside of primary health care, and approximately 25–30 % of specialists working in primary health care have a specialty other than general practice (Finnish Medical Association, unpublished information). Therefore, it might be that other specialists working in primary health care are especially satisfied with their career path. Still, the reasons behind this finding would definitely need some further examination.

For females, motives related to work-family balance were more important, while male respondents preferred motives related to the external factors of work life, such as career, professional appreciation and salary. For females, flexibility and quality of life seem to be important factors when choosing their medical career, even when it means compromising professional achievements [37–41]. On the other hand, differences in controllable lifestyle, on-call work and work-family balance play a more significant role than formerly in a young doctor’s career decisions, also among young male doctors [42–44]. However, according to this study, gender or motives related to a controllable lifestyle do not seem to correlate with satisfaction with the chosen specialty. Still, it is important to take them into consideration when developing the selection process and content of postgraduate medical education for the younger generation of doctors. Nevertheless, the youngest group of respondents seemed to be the most satisfied with their specialty. The reasons for this are not clear. It is possible that members of the younger generation have chosen their career more carefully and truly are more satisfied with their medical specialty. But this may also indicate, for example, that one gets more critical towards own choices in later stages of one’s career.

Perceived quality of the specialist training programme had a very small role in the choice of medical specialty. However, at the same time *Correspondence of the specialist training to the current work* significantly predicted satisfaction with the chosen specialty. It seems that medical educators should be able to better reveal the content of the postgraduate medical training as well as the content of the work as a medical specialist.

The strength of this study is that it provides national data on Finnish working-age medical specialists. However, there are obviously some limitations. First of all, when the first study in this series was conducted in 1988, there were few other studies addressing this issue or requirements to validate the questionnaire. Since then the questionnaires have been largely the same in order to achieve comparability. With questionnaires of this kind, one needs to acknowledge possible bias stemming from the respondents’ self-reporting. In some cases respondents may complete the questionnaire differently when they know the results are going to be seen. Answering *“No”* to the question about choosing the same medical specialty now does not indicate whether the respondent actually intends to seek another specialty. No assumptions can therefore be made in this direction. Instead, our interpretation is that it indicates dissatisfaction with the chosen specialty, and has been used as such in this study. The terms used in the study were not explained in the questionnaire. Therefore, we cannot be absolutely sure how the respondents understood the meaning of, for example, *Diversity of work* as a reason to choose a specialty. Nevertheless, this should not have any major impact on the conclusions of this study.

In this study the respondents had to think back to the time when they were deciding which specialty training programme they would choose and try to remember their reasons at that time. It has been reported that important life events remain fairly well fixed in memory [45]. Since the choice of professional career can be considered such an event, one can assume that items related to it are well recalled.

## Conclusions

As mentioned earlier, there is an urgent need in Finland to evaluate and regulate the number of postgraduate trainees in medical specialty training programmes. However, the real challenge here is to combine this with the fact that under the current situation most specialists are actually quite happy with their choice of specialty. To be satisfied with their careers, doctors should continue to be able to find a speciality that they find interesting and that suits their personality and life [46, 47]. At the same time, the role of chance should be kept as minimal as possible. To do this, career guidance should have a role during medical school and also after graduation [36]. Medical students and graduates should also be kept well informed about the present employment situation in each specialty, so that they can take it into consideration and, if necessary, give thought to some other specialty than their first choice. Also interviews might help in the selection process [48–50]. The importance of first working experiences and the example set by colleagues is something that needs to be looked at closely, especially in specialties that are lacking a sufficient workforce. Furthermore, correspondence to the content of the work as a medical specialist should be the main target when developing the postgraduate medical training to meet the demands of the 21^st^ century.

### Ethics approval and consent to participate

Not applicaple. According to Ethics Committee and based on the Finnish Medical Research Act and Personal Data Act, studies of this kind do not need ethical approval, since they do not affect the respondent’s personal integrity and as respondents are free to choose whether to respond or not. Respondents were fully informed about the use of the questionnaires in the cover letters. Because of this it was presumed that respondents gave an informed consent when choosing to answer the questionnaire.

### Availability of data and materials

The data of this study is available upon request from the authors.
